# Binding of DNA-bending non-histone proteins destabilizes regular 30-nm chromatin structure

**DOI:** 10.1371/journal.pcbi.1005365

**Published:** 2017-01-30

**Authors:** Gaurav Bajpai, Ishutesh Jain, Mandar M. Inamdar, Dibyendu Das, Ranjith Padinhateeri

**Affiliations:** 1 Department of Biosciences and Bioengineering, Indian Institute of Technology Bombay, Mumbai, India; 2 Department of Civil Engineering, Indian Institute of Technology Bombay, Mumbai, India; 3 Department of Physics, Indian Institute of Technology Bombay, Mumbai, India; Rutgers University, UNITED STATES

## Abstract

Why most of the *in vivo* experiments do not find the 30-nm chromatin fiber, well studied *in vitro*, is a puzzle. Two basic physical inputs that are crucial for understanding the structure of the 30-nm fiber are the stiffness of the linker DNA and the relative orientations of the DNA entering/exiting nucleosomes. Based on these inputs we simulate chromatin structure and show that the presence of non-histone proteins, which bind and locally bend linker DNA, destroys any regular higher order structures (e.g., zig-zag). Accounting for the bending geometry of proteins like nhp6 and HMG-B, our theory predicts phase-diagram for the chromatin structure as a function of DNA-bending non-histone protein density and mean linker DNA length. For a wide range of linker lengths, we show that as we vary one parameter, that is, the fraction of bent linker region due to non-histone proteins, the steady-state structure will show a transition from zig-zag to an irregular structure—a structure that is reminiscent of what is observed in experiments recently. Our theory can explain the recent *in vivo* observation of irregular chromatin having co-existence of finite fraction of the next-neighbor (*i* + 2) and neighbor (*i* + 1) nucleosome interactions.

## Author summary

The fate of a cell is not just decided by the genetic code but also by the nature of the 3D organization of the protein-bound DNA, known as chromatin. Chromatin packaging is believed to be in a hierarchical manner, and one of the crucial stages in the packaging is argued to be having a zig-zag structure with specific width of 30 nm. However, most of the recent experiments failed to find any zig-zag-like ordered arrangement of chromatin in living cells. In this work, we address this puzzle, and argue that any regular, ordered, packaging of chromatin is unviable given that certain types of proteins can bind and bend the chromosomal DNA.

## Introduction

In cells, DNA, with the help of a large number of proteins, is packaged into a higher order structure known as chromatin. In eukaryotes, in its simplest level of packaging, DNA is wrapped around histone proteins to form a “beads on a string” form of chromatin having a width ≈ 10 nm. The ‘bead’ here is a nucleosome—147 bp of DNA wrapped around an octamer-complex of histone proteins [1]. When the cells are preparing to divide, one observes that the DNA, with the help of many proteins, assumes a highly condensed structure. It is being thought that the 10-nm string of chromatin is further packaged in a hierarchical manner to produce this highly compact mitotic chromosome [1]. What this hierarchy of structures, if any, is and exactly how a DNA chain gets packaged into this highly compact form are interesting open questions [2–4].

Based on theoretical arguments and a few (mostly *in vitro*) experiments, it was proposed that the 10-nm chromatin string will fold itself into a higher order structure having an approximate width of 30-nm [5–7]. Some of the earliest theoretical models argued that the 30-nm chromatin should form a solenoid structure by coiling the known 10-nm fiber [5, 6]. However, later studies argued that the 10-nm chromatin should rather be arranged in a zig-zag fashion [8–12], which was subsequently supported by high resolution X-ray imaging of crystals made of tetra nucleosomes [13] and other *in vitro* biochemical studies of nucleosome arrays [14]. Since the zig-zag model is compatible with chromatin having a variety of linker DNA lengths, it became the most popular model for higher order structure, in the literature. However, contrary to all theoretical expectations and *in vitro* observations, experimental investigations could not prove the existence of 30-nm fibers *in vivo* [15–19]. Even though some experiments on chicken erythrocyte and starfish sperm chromatin, where non-histone proteins are very low, reported short-range zigzag structure [17, 20], detailed studies of chromatin in other nuclei, using Cryo-EM and synchrotron X-ray scattering methods, argued that the 30-nm structure is non-existent *in vivo*/*in situ* [21, 22]. A set of small angle X-ray scattering experiments concluded that 30-nm fibers are non-existent *in vivo*, and they could not find any particular lengthscale beyond 10 nm [22]. Neither in the interphase nor in the mitotic phase could experiments find any of the expected higher order packaging [21–24]. Why the theoretically predicted, and *in vitro*-observed, 30-nm fiber is elusive *in vivo* is a puzzle in the field of chromatin biology.

**Fig 1:**
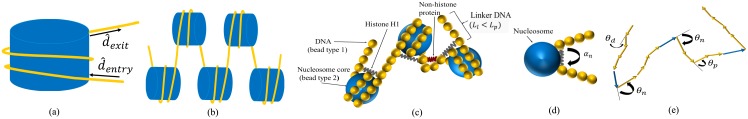
(a) When the DNA (yellow chain) is wrapped around the histone octamer (blue), we can represent the direction of the DNA segments that enters and exits the histone core as two vectors as shown here. The relative orientations of these entry/exit vectors will influence the local structure of the chromatin. (b) Linker DNA is typically rigid and straight. Rigid linker DNA and restricted orientations of entry/exit DNA segments will promote a zig-zag structure. (c–d): Schematic diagram describing the models: (c) Bead-spring model—DNA is modelled as a polymer made of beads of type-1 (yellow). 14 DNA-beads wrap around the histone-octamer bead (type-2, blue) to form a nucleosome. The picture also depicts linker histone H1 constraining entry/exit DNA beads (black spring), non-histone protein bending the linker region (red spring), and rigid linker regions when it is free of non-histone proteins. (d) Equivalently, nucleosome is considered as a bead that constraints the entry/exit DNA segments at an angle *α*_*n*_. (e) FRC model—chromatin is modelled as a long 3D chain of *N* vectors. Yellow vectors represent linker DNA segments, which make a small angle *θ*_*d*_ with respect to its neighbor vector (in the absence of a non-histone protein); blue arrows represent histone-bound DNA having an angle *θ*_*n*_ = *π* − *α*_*n*_ between them. When a non-histone protein is present in the linker region, yellow tangents make a relative angle of *θ*_*p*_ as shown.

In this work, we address this puzzle of the 30-nm structure, based on basic biophysics principles involved in the packaging of chromatin. However, to get into the details, we need to understand the basic logic behind the proposed zig-zag structure of chromatin. We know a few facts: (i) DNA wraps around histone-octamer in nearly two turns such that the entry and exit segments of the DNA make a fairly restricted angle [25, 26] (see Fig 1). (ii) Linker DNA lengths (*L*_*l*_) are in the range 20–60bp. (iii) DNA is a highly stiff elastic chain in the length scale below persistence length (*L*_*p*_ = 150bp) [10, 11, 27].

Since typically *L*_*l*_ < *L*_*p*_, the linker DNA will be rigid and straight. Given that nearly 2 turns around histone-octamer would bring back the DNA to approximately the same side of the nucleosome where it entered, the combination of the two factors—entry/exit angle and stiff linker region—would give rise to a preferred structure like a zig-zag (see Fig 1b). There have been experimental studies that have indicated the occurrence of straight linker regions [10, 11] and restricted entry/exit angles [25, 26] in zig-zag chromatin. All other constituents (ions, low-salt buffer conditions, linker histones) can be thought of as agents influencing these two factors [28–33].

Even though the entry/exit angle and DNA rigidity have been the primary argument supporting zig-zag structure, this neglects a couple of facts: (i) *in vivo*, non-histone proteins can bind on the linker region and distort the orientation. For example, highly abundant high mobility group (HMG) proteins (e.g., nhp6 in yeast), and many other architectural transcription factors are known to bend DNA [34–38]. This bending by non-histone proteins can potentially affect the structure of chromatin. (ii) Since DNA has a nonlinear bending elasticity, it can have flexible hinges [39–43]. Given that DNA bending due to non-histone proteins is a certainty, it is important to consider them in any model that investigates the chromatin structure *in vivo*. Existing models primarily predict zig-zag or solenoid-like structures [44–46]. Recent models also predict polymorphic structures based on the variation in nucleosome repeat lengths [32, 47]. However, none of the existing papers, to the best of our knowledge, have accounted for the effect of local DNA curvatures resulting from non-histone protein binding and/or the possibility of highly flexible regions due to other factors. Neither have these studies systematically investigated how the chromatin structure would appear when a large number of nucleosomes (beyond 100 nucleosomes) are present in the presence of DNA-bending proteins.

In this work we study the higher order folding of nucleosome-bound DNA taking into account the possibility of non-histone proteins binding along the linker region, bending the DNA locally. Performing extensive simulations with two different models for chromatin, we show that (i) in the absence of non-histone protein binding, the nucleosome-bound DNA folds into a regular zig-zag structure; (ii) after the introduction of the non-histone proteins that bend linker DNA, the regular zig-zag structure starts disappearing. We compute the chromatin structure in the presence of specific proteins such as nhp6 and HMG. As a function of the density of bound non-histone proteins, we find that there is a transition from a zig-zag structure to an irregular higher order structure. We also investigate the influence of linker length on the formation of irregular structures.

## Materials and methods

To investigate the role of DNA-bending proteins in higher order chromatin structures, we performed two kinds of simulations: (i) Brownian dynamics (BD) simulations considering a bead-spring model of DNA and accounting for nucleosome and proteins explicitly, and (ii) Monte Carlo (MC) simulations considering a freely rotating chain (FRC) model for chromatin.

### Bead-spring model

In the first model (Fig 1(c)), DNA is considered as a polymer chain of *N* beads of type-1 (small, yellow beads) connected by linear springs of stiffness *k*_*s*_. Bending elasticity of DNA is introduced using the worm like chain model (see details in the following text). The system we simulate has *M* nucleosomes where each nucleosome is created by wrapping 14 beads of DNA (=147 bp) in ≈ 1.7 turns (8 beads per wrap) around a second type of bead (histone core particle bead, blue, Fig 1(c)). The DNA-nucleosome interaction is accounted for by connecting the 14 beads via another set of linear springs having stiffness *k*_*n*_. Linker histones (H1) are modeled as another type of spring connecting DNA segments that enter/exit nucleosomes. Two nucleosomes, when they are closer than a prescribed cut-off distance, can interact with each other; in the model, this inter-nucleosome interactions are also approximated as spring forces between two histone core particles. An important addition in the model is the presence of DNA-bending non-histone proteins—we assume that non-histone proteins can bind in the linker regions and bend the DNA locally. To mimic this, we consider non-histone protein as a spring that will connect two DNA-beads in the middle of the linker region and bring them together (see Fig 1(c), red); the net result is bending of the linker DNA. As mentioned earlier, the stretching energy of DNA (*U*_*s*_), the DNA histone interaction energy (*U*_*n*_), the linker histone interaction energy (*U*_*l*_), the interaction energy of the non-histone protein (*U*_*p*_), and the inter-nucleosome interaction energy (*U*_*h*_) are approximated using a quadratic potential with different stiffnesses (*k*_*α*_) and equilibrium extensions(*r*_*α*_) as:

$$\begin{array}{c} U_{\alpha }=\frac{k_{\alpha }}{2}\sum_{i,j}\left(\right|r_{i}^{\left(\beta \right)}-r_{j}^{\left(\gamma \right)}{\left|-r_{\alpha }\right)}^{2} \end{array}$$ (1)

where *α* ∈ {*s*, *n*, *l*, *p*, *h*}, *β* and *γ* are symbols indicating whether the bead is a DNA-bead ($r_{i}^{\left(1\right)}$) or a nucleosome bead ($r_{i}^{\left(2\right)}$). (i) For DNA chain *α* = *s*, *β* = *γ* = 1, *j* = *i*+1 and *i* ranges from 1 to *N* − 1. (ii) For nucleosome core-DNA interaction *α* = *n*, *β* = 2, *γ* = 1, *i* ranges from 1 to *M* and *j* takes 14 values, for each *i*, representing the identity of the correspoding histone-bound DNA beads. Since nucleosomes are not dynamic in our study (no sliding or disassembly of nucleosomes), we have also prepared the system, equivalently, replacing the nucleosome as an effective bead with $U_{n}=k_{n}^{\text{eff}}\sum_{i}1-\cos(\alpha_{i}-\alpha_{n})$ which constraints the DNA entering and exiting the nucleosome at an angle at *α*_*n*_(see Fig 1(d)) [48]. This saves computational time and allows us to run BD simulations for a longer chromatin. (iii) For linker histone interaction *α* = *l*, *β* and *γ* represent the entry and exit DNA beads, respectively, *j* = *i* and *i* ranges from 1 to *M* − 1. (iv) For non-histone protein binding at the linker region *α* = *p*, *β* and *γ* represent the identity of the location where non-histone proteins bind; *i* goes from 1 to *ν*, *j* = *i* + 3, where *ν* is the total number non-histone proteins bound. Different non-histone proteins will have different *r*_*p*_ values representing the bending angles they induce, as we discuss later in the manuscript (also see Supporting Information (SI) S1 Text and S1 Fig). (v) For inter-nucleosome interaction *α* = *h*, *β* = *γ* = 2; at every instant *i* and *j* are computed such that the *i*^*th*^ nucleosome can interact with the *j*^*th*^ neighbor positioned below a certain cut-off distance = 9*a*. When two nucleosomes are beyond this cut off distance, the inter-nucleosome interaction energy of the pair is zero. Since inter-nucleosome interactions are dominated by histone tails, and since nucleosomes have only finite number of tails, we assume that each nucleosome interacts only with two nearby nucleosomes (*j* ≤ 2) (We have also done simulations accounting for more than two tails). To mimic the steric hindrance of DNA beads, the repulsive part of the standard Lennard-Jones (LJ) potential (*U*_*LJ*_) is used (when $|r_{i}^{\left(1\right)}-r_{j}^{\left(1\right)}|<2a$) such that

$$\begin{array}{c} U_{LJ}=\epsilon \sum_{\begin{array}{l} i=1\\ j>i \end{array}}^{N-1}\left[\frac{{\left(2a\right)}^{12}}{{\left(|r_{i}^{\left(1\right)}-r_{j}^{\left(1\right)}|\right)}^{12}}-2\frac{{\left(2a\right)}^{6}}{{\left(|r_{i}^{\left(1\right)}-r_{j}^{\left(1\right)}|\right)}^{6}}+1\right] \end{array}$$ (2)

where *ϵ* is potential well depth. The energy is zero when $|r_{i}^{\left(1\right)}-r_{j}^{\left(1\right)}|\geq 2a$ The bending energy (*U*_*b*_) of the DNA chain is given by

$$\begin{array}{c} U_{b}=\frac{k_{b}}{2a}\sum_{i=1}^{N-1}\left(1-\cos\theta_{i}\right) \end{array}$$ (3)

where *k*_*b*_ is the bending stiffness of DNA and *θ*_*i*_ is the angle between two nearby bonds in the bead-spring model. The total energy of the chromatin in this model is given by *U*_*tot*_ = *U*_*s*_ + *U*_*LJ*_ + *U*_*b*_ + *U*_*l*_ + *U*_*n*_ + *U*_*p*_ + *U*_*h*_. Since expanding any potential energy close to its stable minimum gives us a quadratic function, we assumed a harmonic nature for most of our interactions. However, we have also done simulations with other functional forms (e.g, Morse potential) to ensure that the results are robust (see S1 Text). The system, accounting for all potentials, is simulated using BD by solving the equation given by

$$\frac{dr_{i}^{\left(\gamma \right)}}{dt}=-\mu_{0}^{\left(\gamma \right)}\;\nabla_{r_{i}^{\left(\gamma \right)}}U_{tot}\left(t\right)+\xi_{i}^{\left(\gamma \right)}\left(t\right)$$ (4)

where *γ* = 1 or 2 represents the identity of the beads, namely the DNA beads and nucleosome core particles, respectively. *μ*_0_ is the mobility, and **ξ**_*i*_ is the thermal noise experienced by each particle such that 〈**ξ**_*i*_〉 = 0 and 〈**ξ**_*i*_(*t*) ⋅ **ξ**_*j*_(*t*′)〉 = 6*k*_*B*_ *Tμ*_0_ *δ*_*ij*_ *δ*(*t* − *t*′) (see [50] for details). Given an initial condition, we solve the equation taking a set of parameters as discussed in the following text. We have also done simulations with standard simulator tool LAMMPS to ensure robustness of our results [51](see S1 Text). From the simulations, we obtain the positions of all beads as a function of time (**r**(*t*)); when the system equilibrates we sample shapes of the chromatin and compute different quantities as discussed in the Results section.

### Parameters used in BD simulation

The basic length scale is taken as one helical repeat of DNA 10.5 bp, which is also the diameter of the DNA bead (2*a*) and the equilibrium distance of the DNA spring (*r*_*s*_). The bending stiffness of DNA is *k*_*b*_ = 50 *k*_*B*_T nm where *T* = 300 K, and *k*_*B*_ is the Boltzmann constant [32]. Since, within this model, we want to hold the interacting DNA nearest-neighbor beads, DNA-nucleosome beads, and DNA-protein spring unstretchable, we took the corresponding stiffness values to be very high: $k_{s}=k_{n}=k_{p}=100k_{B}T/r_{s}^{2}$. We have taken the linker histone interaction constant *k*_*l*_ in the range 30 to $100k_{B}T/r_{s}^{2}$ and $k_{n}^{\text{eff}}=50k_{B}T$. For the purpose of this work, the exact value of these stiffness parameters are irrelevant as long as they are high enough to reduce large fluctuations (see S1 Table). Based on the literature and various geometrical constraints in the problem, we take *r*_*n*_ = 8*r*_*s*_/*π*, *r*_*l*_ = 2.5*r*_*s*_, and *r*_*h*_ = 4.2*r*_*s*_ [32]. We consider effects different specific non-histone proteins by varying value of *r*_*p*_. Since highly abundant proteins like nhp6 (yeast) and HMG-B are known to bend DNA making bend angles of ≈ 90° or ≈ 120° [52, 53], we chose values *r*_*p*_ = 2.4*r*_*s*_ and *r*_*p*_ = 2*r*_*s*_ such that the appropriate angles are formed (see S1 Text for more information). The timescales and mobility parameters are taken as Δ*t* = 0.04ns, $\mu_{0}^{\left(1\right)}=2\times 10^{-4}r_{s}/k_{B}T\Delta t$, and $\mu_{0}^{\left(2\right)}=1.5\times 10^{-4}r_{s}/k_{B}T\Delta t$ respectively (see S2 Table for details on non-dimensionalization of some of these parameters). We run the simulations for a time (0.04s) which is much longer than the time it takes for the system to reach a steady state (milliseconds) having constant mean energy and radius of gyration.

### FRC model

To understand how chromatin is structured *in vivo*, one needs to simulate a long chromatin having a large number of nucleosomes (thousands of nucleosomes) in a large parameter space. Since it is computationally intensive to do so using BD simulations (typical BD simulations simulate <100 nucleosomes), a freely rotating chain model for the chromatin is employed. In the FRC model, we consider chromatin as a long 3D chain made of *N* tangent vectors. Orientations of the vectors are chosen such that the angle between the vector *i* and *i* − 1 is *θ*_*i*_ (see Fig 1(e)). If the *i*^*th*^ vector is a DNA vector, it makes a relative angle of $\theta_{i}=\sqrt{4a/L_{p}}$ with its neighbor [54]. As per the definition of FRC, the rotation angle *ϕ* is chosen randomly [54]. From a structural point of view, it can be argued that binding of the histone-complex (or any protein) constrains DNA subunits entering and exiting the histone octamer (protein) [26]. Therefore, if the *i*^*th*^ bead in the FRC is a nucleosome, a relative angle of $\theta_{i}=\frac{2\pi }{3}$ is assigned between the two adjacent tangents [25]. The corresponding *ϕ*_*i*_ angle is chosen in a range of −10° to 10° randomly so as to satisfy the known zig-zag structure seen in earlier simulations. We also introduced the DNA-bending non-histone proteins in the FRC as follows. While constructing the FRC, at every linker region, one neighboring vector pair in the middle is chosen with a given probability *ρ*, and imposed a certain angle *θ*_*p*_ representing the binding activity of non-histone proteins. First we consider bending due to specific proteins like nhp6 and HMG-B (*θ*_*p*_ ≈ 90° and 120°) [52, 53] and then we do a calculations considering a distribution of angles around these typical values. The angle *ϕ* here is chosen randomly between −10° and 10°. In this manner, we constructed a large number of chromatin configurations (many realisations), each having 2000 nucleosomes. To quantify the structure of these configurations we calculated *I*(*k*) which is the probability that any nucleosome is in “contact” with its *k*^*th*^ neighbor [32] (see S2 Text for details). Two nucleosomes are defined to be in contact when the distance between them is below a cut-off distance of 16 nm (9*a*), which is the estimated mean length of histone tail interactions.

## Results

**Fig 2:**
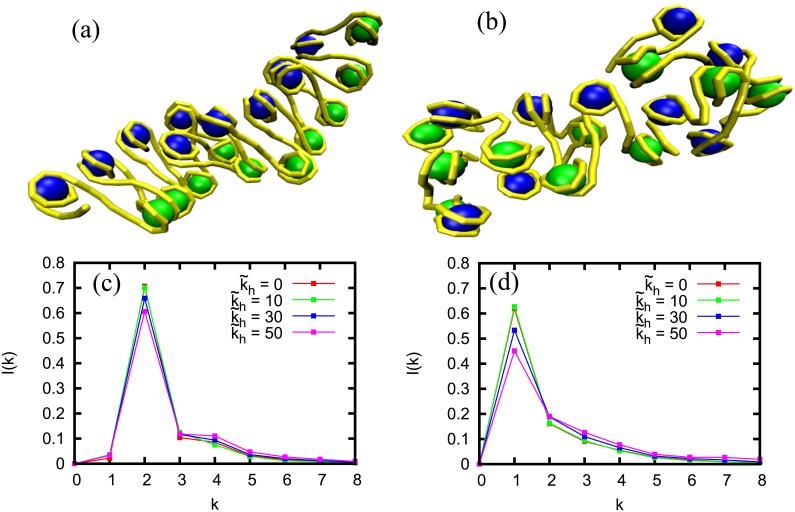
(a–b): Snapshots of chromatin from BD simulations showing DNA (yellow) and nucleosomes (blue and green) [55]. (a) In the absence of non-histone proteins; a zig-zag structure is seen. (b) In the presence of non-histone proteins that bend linker DNA (density *ρ* = 0.5). DNA-bending brings neighbouring nucleosomes closer to each other mixing the blue and green. This destroys the zig-zag nature where, typically, next neighbors (same-color nucleosomes) are closer than the neighbors (different colors). Both have inter-nucleosome interaction with ${\tilde{k}}_{h}=50$. (c–d): *I*(*k*) from BD simulations. (c) Without (${\tilde{k}}_{h}=0$) and with inter-nucleosome interactions (${\tilde{k}}_{h}=10,30,50$) and in the absence of any non-histone protein. Here *I*(*k*) peaks at *k* = 2 indicating the formation of zig-zag structure for each ${\tilde{k}}_{h}$. (d) Without (${\tilde{k}}_{h}=0$) and with inter-nucleosome interactions (${\tilde{k}}_{h}=10,30,50$) in the presence of non-histone proteins(*ρ* = 0.5). Here the peaks at *I*(1) imply that neighboring nucleosomes are geometrically close to each other, and the zig-zag structure is dismantled in the presence of non-histone proteins.

First, we present the results from BD simulations. In Fig 2, we present the structure of chromatin (3D positions of nucleosomes and linker DNA) having M = 20 nucleosomes, and uniform linker length of 42 bp. In the absence of any non-histone proteins that bend the linker region, we get a nice zig-zag structure (Fig 2a; also see S2 Fig). For convenience, we used a color code by which one can see how odd (blue) and even (green) nucleosomes are nicely separated in a zig-zag fashion such that the *i*^*th*^ nucleosome is close to the (*i* + 2)^*nd*^ nucleosome (Fig 2a). However, once we introduce some amount of non-histone proteins that bind on the linker region and bend it locally(*ρ* = 0.5), the zig-zag structure is destroyed and an irregular structure emerges. Note that the colors are now mixed indicating the loss of any regular arrangement.

To characterize these structures, we computed the corresponding contact probabilities *I*(*k*) [32]. In the absence of any non-histone protein binding, *I*(*k*) peaks at *k* = 2 (Fig 2c). This means that most of the nucleosomes are in contact with their next neighbor. This is the signature of a typical zig-zag structure. Given that linker DNA regions are nearly straight, the zig-zag structure is maintained for various values of inter-nucleosome interaction parameters—different curves represent different inter-nucleosome interaction parameter (non-dimensionalized ${\tilde{k}}_{h}=2k_{h}a^{2}/k_{B}T$) values. We then computed *I*(*k*) in the presence of non-histone proteins that distort linker DNA. The results are shown in Fig 2d for different inter-nucleosome interactions and 50% density of bound non-histone proteins. The *I*(*k*) is no more peaked at *k* = 2; the peak is shifted to *k* = 1, indicating the destruction of the zig-zag structure. For higher inter-nucleosome interactions (different curves), two nucleosomes come very close and the overall structure is more tightly packed. The peak probability decreases slightly with higher interactions as more and more far away neighbors get locked within the cut off distance. These results indicate that the presence of DNA-bending non-histone proteins can be a crucial factor in determining the chromatin structure at the length scale of a gene, and may plausibly explain why the 30-nm higher order structure is elusive under in vivo conditions (also see S3 and S4 Figs). To test whether the precise model for linker histone would affect our results, we did a set of simulations considering linker histone as a bead that interacts with two entry/exit linker DNA and the corresponding nucleosomes as suggested by data in the literature [49] (see S1 Text). Results in S5 Fig show that this does not change our findings. We also did BD simulations considering nucleosomes as angle-inducing proteins (Fig 1(d); see the following text). This also gives us similar results as above.

### Specific proteins with different DNA-bending angles and sizes

**Fig 3:**
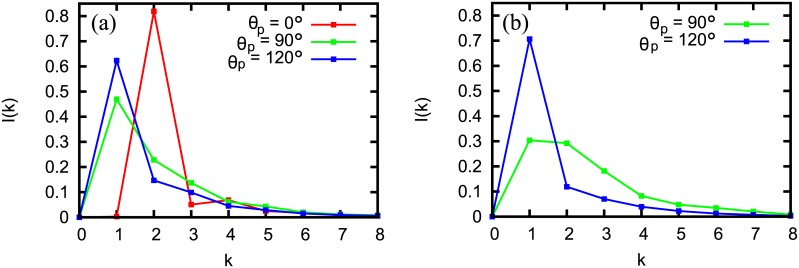
BD simulation with two specific DNA-bending geometries corresponding nhp6 and HMG-B. (a) *I*(*k*) for proteins having a size of 20bp (one angle involving 2 bonds in the model) with bending angle = 120° (blue) and 90° (green). The red curve is the control when there is no DNA-bending protein. (b) For proteins having a size of 30bp (one angle involving 3 bonds in the model; see S1 Text). The plots are for ${\tilde{k}}_{h}=50$ and *ρ* = 0.5 and with nucleosomes as angles *α*_*n*_ = 60° done using LAMMPS [50]. In all the cases, the presence of DNA-bending proteins shifts the peak away from *k* = 2 indicating the destruction of zig-zag.

We investigated the role of specific DNA-bending proteins in determining the structure of chromatin using the above BD simulations. DNA-bending protein in yeast nhp6 is reported to bend DNA making angles of *θ*_*p*_ ≈ 120° [52, 56] or ≈ 90° [52, 57]. The HMG-B protein is reported to bend DNA making *θ*_*p*_ = 90° [53, 58]. The proteins are typically known to have a size (footprint on DNA) of ≈ 20*bp* (HMG-B, nhp6; bend involving 2 bonds) or ≈ 30bp (some proteins in the HMG family; bend involving 3 bonds) [58, 59]. In Fig 3 we present our results in the presence of nhp6 and HMG-B proteins bending DNA with angles *θ*_*p*_ = 90° and 120°. In the absence of any protein(*θ*_*p*_ = 0), I(k) peaks at *k* = 2 indicating zig-zag (Fig 3(a)). When we introduce nhp6 or HMG-B having DNA-bending angle of 90°, size = 20bp, we get a peak at *k* = 1 (green curve). The peak at *k* = 1 becomes more dominant if the bending angle increases to 120° (blue curve). For larger proteins (size 30bp) too we see that the zig-zag structure is destroyed (Fig 3(b), peak is not at *I*(2)). All the above results are for a protein density of *ρ* = 0.5 per linker region, which is close to the known abundance of the DNA-bending non-histone proteins [35]. see S6, S7 and S8 Figs for more angles and parameters. We also performed simulations with different types of inter-nucleosome interactions by varying the strength of the interactions and the nature of the potential (see S3, S4 and S9 Figs).

**Fig 4:**
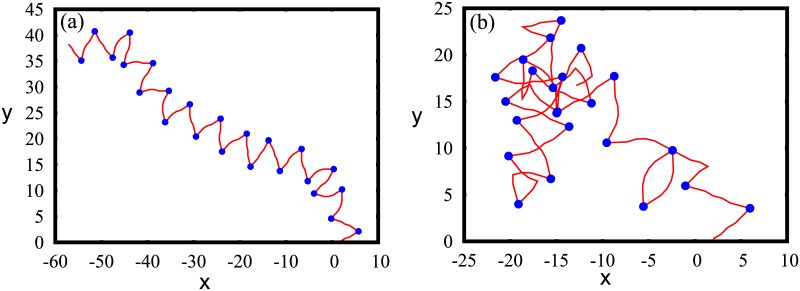
Snapshots of chromatin simulated using the FRC model in 2D showing linker DNA (red) and nucleosomes (blue). The linker length is ≈ 42 bp. (a)In the absence of any non-histone protein, we get a nice zig-zag-like structure. (b)In the presence of non-histone proteins where each linker region has a probability of 0.24 for non-histone protein to bind. The presence of non-histone proteins is modeled as a bend in the linker region. The non-histone proteins (not marked separately) are visible as sharp angles between two neighboring blue dots.

To get an intuitive understanding of the above results, we performed simple 2D simulations using the FRC model as discussed in the text earlier. We simulated the structure of a chromatin having 24 nucleosomes, with uniform linker length of 42 bp, and the results (XY position nucleosomes and linker DNA) are plotted in Fig 4. In the absence of non-histone proteins, the structure is a clear zig-zag (Fig 4a). When we add non-histone proteins that bind along the linker region and bend the linker DNA, beyond a critical density of bound non-histone proteins, we completely lose the zig-zag nature and obtain a chromatin with irregular structure (Fig 4b, *ρ* = 0.24, *θ*_*p*_ = 120° with random orientations). This simple numerical study supports the hypothesis that the role of non-histone proteins is crucial in deciding the higher order structure of chromatin.

### Large-scale chromatin organization

**Fig 5:**
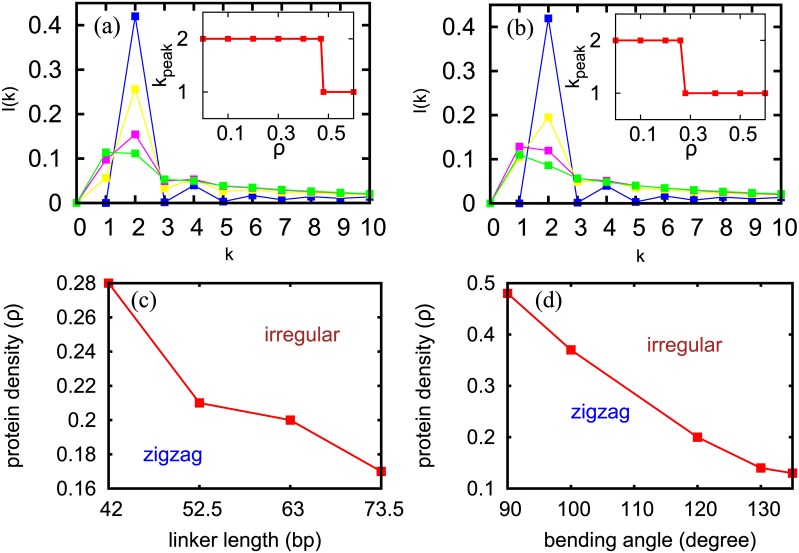
From 3D FRC model with 2000 nucleosomes. (a) *I*(*k*) without any non-histone proteins (blue curve) and with different densities of non-histone proteins: *ρ* = 0.1 (yellow), *ρ* = 0.3 (pink), and *ρ* = 0.5 (green). The peak at *k* = 2 is shifted to *k* = 1 on increasing *ρ*. Here, the bending angle of the non-histone protein is *θ*_*p*_ = 90° representing nhp6 and HMG-B. Inset: the peak location (*k* value at which *I*(*k*) is peaked), *k*_*peak*_, for different *ρ*. This indicates a transition from a zig-zag to an irregular structure. (b) The same as (a) but with *θ*_*p*_ chosen randomly from 90°–135° (c) A phase diagram obtained by varying the linker length and the density of proteins in the linker region (*θ*_*p*_ as in (b)). The red dots represent the values of the parameters at which the transition from zig-zag to irregular structure happens (i.e., parameter values at which *I*(1) = *I*(2)). (d) A similar phase diagram obtained by varying the bending angle (representing different proteins) and the protein density (linker length = 42*bp*).

So far we did simulations with a small number of nucleosomes (*M* ≈ 20, approximate length scale of a single gene). However, what will be the chromatin organisation in the longer length-scale (length scale of many genes) accounting for a large number of nucleosomes is an important question. Since performing BD for large systems is computationally expensive, and since both BD and FRC simulations give similar results, we implemented the 3D FRC model to probe large-scale organization of chromatin. Using the FRC model, we generated a large number of (≈ 10^7^) equilibrium configurations of chromatin, each having 2000 nucleosomes, both with and without non-histone proteins. Systematically varying protein density (*ρ*), we investigated the amount of non-histone proteins required for the appearance of an irregular structure. To compare the 3D FRC model with and without proteins and to quantify the resulting nucleosome organization in space, here too we computed I(k) (Fig 5a). In the absence of non-histone proteins (Fig 5a, blue), the peak is at *k* = 2 implying a zig-zag structure. As protein density (*ρ*) increases, the *k* = 2 becomes less probable, and the probability of finding *k* ≠ 2 increases. Here the bending angle of non-histone protein is chosen as *θ*_*p*_ = 90° representing proteins like HMG-B or nhp6 [52, 53].

Results presented in Fig 5a suggest that, as a function of one parameter—non-histone protein density—there exists a transition from a zig-zag structure (dominant peak is at *k* = 2) to an irregular structure (dominant peak at *k* ≠ 2). The plot of peak position (the value of *k* at which *I*(*k*) peaks) as a function of protein density (Fig 5a, inset) captures this transition and shows that the transition happens when the protein density is ≈0.48. This is also the parameter regime where *I*(1) is comparable to *I*(2)—this is important since recent experiments have indicated that chromatin has an irregular structure, and at the same time there exist both *i*/*i* + 1 and *i*/*i* + 2 contacts [60]. Given that, in reality, there are proteins of different types binding in a heterogeneous manner (e.g. LEF1: 107°-127°, nhp6: 120°) we repeated the calculation for different set of bending angles—angles randomly chosen from a range between 90° and 135° [56, 59]. The results are shown in Fig 5b. Here too the inset shows a transition from zig-zag to irregular structure as a function of protein density. In both the figures here, the chromatin becomes irregular when *ρ* < 0.5(at *ρ* = 0.48 in Fig 5a and *ρ* = 0.28 in Fig 5b). This is equivalent to having one non-histone protein for every four nucleosomes (≈ 1/0.28) or two nucleosomes (≈ 1/0.48), consistent with abundance of non-histone proteins in the cell [35]. It is known that there exists roughly one HMG protein for every two nucleosomes [35] and hence such irregular structures can be expected for chromatin in which nearly all available HMGB proteins bind at linker regions.

### Phase diagram: Protein density, diversity and linker lengths

Even though the typical chromatin linker length (*L*_*l*_) is rarely larger than the persistence length (*L*_*p*_), it has been argued that the linker length variation may affect the structure of chromatin [32, 47, 61, 62]. We did FRC simulations for chromatin having different *L*_*l*_ values (see S10 Fig). For large *L*_*l*_, *I*(2) decreases considerably; however given that the largest biologically relevant *L*_*l*_ values (≈ 70 bp) are much smaller than the *L*_*p*_ of DNA, *I*(2) still remains dominant. We find that as *L*_*l*_ increases the *ρ* required to create irregular structures becomes smaller. We systematically investigated how the two variables—non-histone protein density and linker length—decide the structure of the chromatin. The result is summarized in a phase diagram shown in Fig 5c. For every value of linker length, we have a “critical” protein density beyond which the chromatin structure is irregular. This prediction can be tested *in vitro* by varying nucleosome density and protein density appropriately. We also did simulations of chromatin having non-uniform linker lengths (*L*_*l*_ taken from a distribution), and it also gave similar results (see S3 Text and S11 Fig). We then computed how different types of proteins, having different bending angles, will affect the phase diagram (see Fig 5d). We varied the bending angles in the range of 90°–135° (see S12 Fig), since this is the range in which most of the relevant non-histone proteins bend DNA. As expected, the more the bending angle, the less the density of protein that is required to make the chromatin structure irregular.

We studied a few other aspects as well: (i) Varied the angle induced by the nucleosomes (*α*_*n*_) and examined its effect (see S13 Fig). (ii)Examined whether the zig-zag or non-histone protein-bound chromatin have a fractal nature [22, 63, 64], and how non-histone proteins would affect packing ratio (*R*_*g*_) (see S4 Text and S14 Fig). (iii) Varied the histone tail interactions (the number of interacting tails and strength) and found how nucleosome contact map might alter in highly interacting regions (heterochromatin) vs loosely interacting regions (euchromatin) (see S2, S3 and S4 Figs).

## Discussion

In this work we examined the role of DNA-bending non-histone proteins in determining the higher order structure of chromatin. Using computer simulations we show that the presence of non-histone proteins will destroy any regular structure of chromatin. We studied a few specific cases of proteins (e.g., HMG, nhp6) that bend DNA by sharp angles in the range 90°–135°. Our study suggests that the structure of chromatin, at the length scale of a few genes (kilobases to megabases) in particular, will be influenced by non-histone proteins that bind and bend DNA. We quantified the influence by computing neighbor contact probabilities for various parameter values.

Given that various DNA-bending proteins are abundant in nearly all cell types, it is imperative that the role of non-histone proteins be studied in detail for understanding the higher order structure. Estimates show that the total number of DNA-bending proteins in the HMG family alone will be roughly half the number of nucleosomes present in the cell [35]. This implies that half of the linker region could be potentially bent. Our study suggests that even when 28% to 48% (depending on the types of proteins present) of the linker regions are bent, the regular 30 nm structure will be destroyed, and we expect to see an irregular structure. A direct prediction of our study is that, even though the regular order is destroyed, there will be a good fraction of nucleosomes that would maintain contacts either with neighbors (*i*/*i* + 1 contacts) or with next neighbors (*i*/*i* + 2 contacts) (see Fig 5). This may be one way of differentiating the irregularity in chromatin structure arising from DNA-bending when compared to the irregularity that may arise due to other reasons such as crowding. Recent Micro-C experiments [60] point precisely in this direction—absence of regular structure with existence of *i*/*i* + 1 and *i*/*i* + 2 contacts.

Our use of a coarse-grained description—FRC model—has an advantage that we do not have to use any microscopic parameters that are difficult to measure. We have only limited number of parameters: entry/exit angle that a nucleosome makes, typical linker lengths, range of angles over which the DNA is bent by non-histone proteins, and the density of non-histone proteins. Since we have some rough estimates of the first three parameters, the only one parameter we have to systematically vary is the density of non-histone proteins. Our results show that irrespective of the precise value of other parameters, chromatin becomes irregular once there are bound non-histone proteins above a critical density in the range ≈ 0.28–0.48 per linker region.

In this work we have assumed that non-histone proteins bind along the DNA with a certain probability (equivalently certain density). It is an interesting question how the results would get affected if the dynamics of the proteins are accounted [65]. Since binding and dissociation rates would add more parameters into the model, a simulation with non-histone protein dynamics is beyond the scope of the current work. We also do not know much about the binding specificity of these non-histone proteins. Hence it is also open to investigate how the results might alter if non-histone proteins like HMG have specific affinity to certain sequences. We have also not accounted for explicit molecular structure of non-histone proteins and linker histones [49] in the model. Since the computational cost of including various molecular details will be very high, a study of a long chromatin with many nucleosomes will only be possible with coarse-grained descriptions as we have done here. We hope future studies would tell us how much molecular details are crucial and how much of coarse-graining can be done without actually affecting the observable behaviour of the system.

### Suggestion for new experiments to test our prediction

Our predictions may be tested in a few different ways. One may perform an *in vitro* experiment reconstituting chromatin in the presence of DNA bending proteins [13, 14, 66, 67]. The DNA-bending proteins are expected to destroy any regular zig-zag structure otherwise seen in the absence of non-histone proteins. Another way would be to perform Micro-C experiments (like Hseig et al [60]) by mutating DNA-bending proteins like nhp6. The prediction is that, when the dominant DNA-bending proteins are mutated, the next neighbour contact probability value will increase. One may also map the positions of DNA-bending proteins such as HMG and correlate them with Micro-C (Hi-C) data so that one obtains a better picture of how the non-histone proteins influence the 3D chromatin structure.

To conclude, in this work, we indicated the importance of non-histone proteins in determining the higher order structure and quantified the contribution of DNA-bending in generating irregular structures. We show that non-histone protein binding with biologically relevant proportions can create chromatin structures with equal fraction of next-neighbor (*i* + 2) and neighbor (*i* + 1) nucleosome interactions, as observed in recent experiments. Our work provides insights into understanding local chromatin structure (in the length scale of a few genes), which is crucial for studying phenomena like histone-modification spreading, accessibility of regulatory regions, and gene regulation in general. We must also acknowledge the role of other factors: in reality, the structure of chromatin will be determined not just by the non-histone proteins but by an interplay between different factors such as crowding, linker length variations, concentration of various ions (e.g Mg++), DNA-bending proteins, and other constituents [30–32]. We hope that this work will trigger more computational and experimental studies in future to probe this aspect more quantitatively, and delineate the relative contributions of all these factors in great detail.

## Supporting information

S1 Text Model. In this test we describe the model that considers nucleosomes as angle-inducing proteins. We also describe model for different sizes of non-histone proteins in this text.
(PDF)

S2 Text Contact probability *I*(*k*). This text describes definition and formula of contact probability.
(PDF)

S3 Text Probability distribution formula for variable DNA linker length. (PDF)

S4 Text Chromatin: An irregular structure with fractal nature?. (PDF)

S1 Table BD: Parameter description. (PDF)

S2 Table Non-dimensional parameters. (PDF)

S1 Fig BD: different non-histone proteins bending DNA. Straight linker DNA, and linker DNA bent by proteins. Some non-histone proteins make angle between 2 bonds (size ≈ 20*bp*) and some non-histone proteins make angle between 3 bonds (size ≈ 30*bp*). Note that since our basic bond sizes are 10.5 bp (helical repeat), we do not have resolution below this length-scale.
(EPS)

S2 FigChromatin configurations varying protein density and inter-nucleosomes strength.Snapshots of chromatin configurations for different parameters; (a), (b), (c) and (d): in the absence of non-histone proteins with non-dimensionalized inter-nucleosome interaction parameter ${\tilde{k}}_{h}=0,10,30$ and 50 respectively; (e), (f), (g) and (h): in the presence of non-histone proteins—25% of the linker regions are bent due the binding of non-histone proteins. Similar to the case above, inter-nucleosome interaction are ${\tilde{k}}_{h}=0,10,30$ and 50 respectively; (i), (j), (k) and (l): in the presence of non-histone proteins—50% of the linker regions are bent due to the binding of non-histone proteins. Similar to the case above, inter-nucleosome interaction are ${\tilde{k}}_{h}=0,10,30$ and 50 respectively. One can see that the zig-zag nature is destroyed as we have more non-histone proteins.(EPS)

S3 FigContact probability *I*(*k*) with high linker histone interaction.(a) *I*(*k*) for high linker histone interaction ${\tilde{k}}_{l}=100$, in the absence of non-histone proteins with inter-nucleosome interaction prameter ${\tilde{k}}_{h}=0,10,30$ and 50. (b) and (c) *I*(*k*), in the presence of non-histone proteins—25% and 50% of the linker regions are bent due to the binding of non-histone proteins respectively.(EPS)

S4 FigContact probability considering 4 interacting histone tails.(a) *I*(*k*), with inter-nucleosome interactions (${\tilde{k}}_{h}=50$) where we vary the number of interacting histone tails (2 or 4 interacting tails for each nucleosome); fig (a) is in the absence of any non-histone protein. (b) The same in the presence of non-histone proteins binding and bending 50% of DNA linker regions. As the number of interacting tails increase, each nucleosome can interact with more neighbors.(EPS)

S5 Fig3D configurations and contact probabilities considering linker histones explicitly.(a) Snapshot of chromatin structure (zigzag) in the absence of non-histone proteins, with inter-nucleosome interactions (${\tilde{k}}_{h}=50$). (b) Snapshot of chromatin structure in the presence of non-histone proteins binding 50% of DNA linker regions with inter-nucleosome interactions (${\tilde{k}}_{h}=50$). (c) *I*(*k*) in the absence of any non-histone protein. (d) The same in the presence of non-histone proteins binding and bending 50% of DNA linker regions.(EPS)

S6 Fig3D configurations and contact probabilities considering nucleosomes as angle-inducing proteins.(a) Snapshot of chromatin structure (zigzag) in the absence of non-histone proteins, with inter-nucleosome interactions (${\tilde{k}}_{h}=50$). (b) Snapshot of chromatin structure in the presence of non-histone proteins binding 50% of DNA linker regions with inter-nucleosome interactions (${\tilde{k}}_{h}=50$). (c) *I*(*k*) without (${\tilde{k}}_{h}=0$) and with inter-nucleosome interactions (${\tilde{k}}_{h}=10,30,50$) and in the absence of any non-histone protein (d) Non-histone proteins bend linker region with *θ*_*p*_ = 120° with (${\tilde{k}}_{h}=0,10,30,50$). Non-histone density is 0.5—that is they bind 50% of DNA linker regions. (e) Non-histone proteins bend linker region with *θ*_*p*_ = 90° with (${\tilde{k}}_{h}=0,10,30,50$). Non-histone density is 0.5—that is they bind 50% of DNA linker regions. All the results are for the case where proteins make angle involving two bonds (i.e., protein size≈ 20bp; see Model section above).(EPS)

S7 FigContact probability *I*(*k*) for different non-histone proteins having different bending angles.Non-histone proteins bend linker region with different angles *θ*_*p*_ = 60°, 90°, 120°, 130°, 135° and 150° with (${\tilde{k}}_{h}=50$). Non-histone density is 0.5. All the results are for the case where proteins make angle involving two bonds(i.e., protein size≈ 20bp; see Model section above).(EPS)

S8 FigNon-histone proteins having different bending angles with a longer size.*I*(*k*) with non-histone proteins that make angle involving three bonds(i.e., protein size≈ 30bp; see Model section above) (a) non-histone proteins bend linker region ${\tilde{r}}_{p}=2r_{s}$ (equivalent to *θ*_*p*_ = 120°) with (${\tilde{k}}_{h}=0,10,30,50$). (b) The same for ${\tilde{r}}_{p}=2.4r_{s}$ (equivalent to *θ*_*p*_ = 90°) with (${\tilde{k}}_{h}=0,10,30,50$). All for the case of non-histone proteins binding 50% of DNA linker regions (i. e. protein density *ρ* = 0.5). Compare these with *I*(*k*) in S6 Fig for a similar case with proteins of a smaller size.(EPS)

S9 FigUsing Morse potential for inter-nucleosome interactions.*I*(*k*), with inter-nucleosome interactions using harmonic (${\tilde{k}}_{h}=50$) and Morse potentials with comparable parameters. (a) *I*(*k*) in the absence of any non-histone protein (b) The same in the presence of non-histone proteins binding 50% of DNA linker regions. See the Model part in S1 Text; Morse potential parameters are chosen such that ${\tilde{k}}_{h}=50$ matches with spring constant of the harmonic interaction.(EPS)

S10 Fig Contact probability *I*(*k*) for different DNA-bending angles. Results from 3D FRC model (M = 2000 nucleosomes) for specific bending angles (a) 100° (b) 120° (c) 130° (d) 135°. Inset in all figures: curve between probability (density) of non-histone protein binding (*ρ*) and k at which *I*(*k*) is peaking (*k*_*peak*_), indicating the transition from zig-zag to an irregular structure.
(EPS)

S11 Fig Contact probability *I*(*k*) for different DNA linker lengths. Results from 3D FRC model (M = 2000 nucleosomes) with and without non-histone proteins for different linker DNA lengths (a), (b), (c), and (d): Main figures depict I(k) for linker lengths 42 bp, 52.5 bp, 63 bp, and 73.5 bp, respectively. The blue curves are the results obtained without any non-histone proteins. Other curves are for different non-histone protein densities given by *ρ* = 0.1 (yellow), *ρ* = 0.25 (pink), and *ρ* = 0.5 (green). Inset in all figures: curve between probability (density) of non-histone protein binding (*ρ*) and k at which *I*(*k*) is peaking (*k*_*peak*_), indicating the transition from zig-zag to an irregular structure as discussed in the main text. In all these figures, bending angles are chosen randomly between 90 and 135 degrees. Different linker lengths may arise due to heterogeneity; nucleosome free regions may also be formed with the help of enzymes that bind and remove nucleosomes.
(EPS)

S12 Fig Structure and contact probability *I*(*k*) for variable DNA linker length. (a) Configuration of chromatin (M = 50 nucleosomes) with variable linker length (31.5 bp to 73.5 bp) such that the average linker length is 42bp; note the zig-zag nature of the structure. (b) Configuration of chromatin in the presence of non-histone protein bound in 25% of the linker regions with variable linker length. Both these configurations are plotted from the FRC data (angle randomly selected from a uniform distribution between 90–135 degrees). (c) Calculation of *I*(*k*) (M = 2000 nucleosomes) for variable linker length (31.5 bp to 73.5 bp; average 42bp) without any bound non-histone protein (blue curve) and with different probability of having non-histone proteins: *ρ* = 0.1 (yellow), *ρ* = 0.25 (pink), and *ρ* = 0.5 (green).
(EPS)

S13 Fig Effect of nucleosome-angle (*α*_*n*_) on chromatin structure. Certain proteins (H1 or other nucleosome binding proteins) may influence the DNA entry/exit angle (*α*_*n*_; see model section above) induced by nucleosomes. Here we plot the peak value of *I*(*k*) on changing *α*_*n*_. Zigzag is destroyed for large nucleosome’s angle.
(EPS)

S14 Fig End to end distances and radius of gyration of chromatin as a function of contour length. (a) RMS end-to-end distance (in bp) as a function of the total number of vectors (links) in the 3D FRC chromatin, for two cases: without (*ρ* = 0) and with (*ρ* = 0.3) non-histone proteins. The red lines represent *R*_rms_ ∝ *N* and the green lines represent *R*_rms_ ∝ *N*^0.5^. The results suggests that non-histone proteins make the chromatin irregular even at very small length scales. (b) Radius of gyration (*R*_*g*_ in bp) as a function of number of nucleosomes (*M*). It is interesting to note that non-histone proteins reduce *R*_*g*_ and hence increases the packing ratio of the chromatin.
(EPS)
