# Supplementary material for: Binding of DNA-bending non-histone proteins destabilizes regular 30-nm chromatin structure
Source: PLoS Comput Biol. 2017 Jan 30;13(1):e1005365. doi: 10.1371/journal.pcbi.1005365 (PMC5305278; doi:10.1371/journal.pcbi.1005365)
Supplement: S1 Text — In this test we describe the model that considers nucleosomes as angle-inducing proteins. We also describe model for different sizes of non-histone proteins in this text. (PDF) [file pcbi.1005365.s001.pdf]

S1 Text. Model

1. Brownian Dynamics considering nucleosomes as angle-inducing proteins

Here we will describe the model that considers nucleosomes as angle-inducing proteins. We considered an effective nucleosome bead of size 11.7nm in diameter, which is equal to the diameter of a nucleosome (core histone with wrapped DNA). We connected this effective nucleosome bead after every 4 DNA beads. We also connected DNA entering and exiting using a spring mimicking H1 histone binding(similar to what is described in the main text). Total stretching energy of the system is calculated from the quadratic potential (spring) with the following equilibrium distances: DNA-DNA bead equilibrium distance = r_s; between DNA-effective nucleosome bead equilibrium distance = $2.2r_s$ and H1 spring equilibrium distance $2.2r_s$. Every effective nucleosome beads make an angle α_n with DNA entering and exiting beads and energy is calculated by

$$U_n = k_n^{\mathrm{eff}} \sum_{i=1}^{M} 1 - \cos(\alpha_i - \alpha_n) \tag{1}$$

where α_i is the angle at the ith location, M is no. of nucleosomes, k_n^{eff} elastic constant which is equal to $50k_BT$ and $\alpha_n = 60°$. Dihedral angles between every nucleosome beads are taken $-10°$ and $10°$ alternatively. Other energies like Bending energy of linker DNA, Lennard-Jones potential energy, histone-tails interaction energy are taken same as our previous model.

2. BD: different non-histone proteins bending DNA

In our model we have distinguished the bending effect of different non-nucleosomal proteins. In yeast, nhp6 is one of the major DNA-bending proteins. It is known that nhp6 creates sharp bends at linker regions and there are two reported angles in the literature. One is 90° [1] and the other one is around 120° [2]. We did simulations for both the cases. For TBP the bending angle is 100° [3]. For LEF-HMG the reported angle is around 110° [4]. Other reported angles vary around these values upto 135°. We have considered HMG proteins of different sizes (footprint) of $\approx 18bp$ and 27bp respectively [5]. Since 20 bp, in our model, correspond to 2 bonds 30bp corresponding to 3 bonds, we implemented this in the following way; we considered (i) angle involving 2 bonds, and (ii) angle involving 3 bonds (see S1 Fig.). (i) In angle involving 2 bonds, potential energy of nhp binding at the linker region is calculated by equation

$$U_n = k_n^{\mathrm{eff}} \sum_{i} 1 - \cos(\alpha_i - \alpha_n) \tag{2}$$

where α_i is the angle created by the ith protein with equilibrium angle being α_n. (ii) In angle involving 3 bonds, we introduce a spring such that the DNA is bent. For known bending angle θ_p, the spring equilibrium distance r_p can be calculated as

$$r_p = r_s + 2\, r_s\, \cos\left(\frac{\theta_p}{2}\right) \tag{3}$$

Note: $\alpha_p = 180° - \theta_p$ and θ_p is the bending angle of the non-histone proteins.

3. Rationale for considering harmonic potential

We have not explicitly considered interactions such as electrostatic interactions and instead approximated most of our interactions as harmonic in nature. The reason we considered harmonic potential is the following. When we consider interactions between physical particles, typically, it will have a repulsive part (that includes steric repulsion) and an attractive part. Whatever be the source of these interactions, the net effect is that it will have a preferred (equilibrium) distance. If

we expand the energy around this equilibrium state, one can approximate the interaction using a harmonic potential. This, of course, is an approximation. Given that our focus is to understand the influence of non-histone proteins, we thought that this approximation is reasonable. However, below, we discuss simulations with other potentials such as Morse potential that mimics realistic interactions.

We also note that, under physiological conditions, the electrostatic interactions are typically screened beyond a length of $\approx$ 1nm, and hence the long range effects can be neglected.

4. Inter-nucleosome interaction using Morse potential

To ensure results are consistent irrespective of the precise function of the potential, we also used Morse potential for inter-nucleosome interactions. Morse potential is calculated by

$$U_h = D_h \sum_{i,j} 1 - \left(\mathrm{e}^{-a\left(|\mathbf{r}_i^{(2)} - \mathbf{r}_j^{(2)}| - r_h\right)} \right)^2 \tag{4}$$

where D_h and a are morse potential constants. These constants are related by a relation $a = \sqrt{\frac{k_h}{2\,D_h}}$ where D_h is taken $1k_BT$.

5. BD simulation using the LAMMPS simulator

To make sure that our results are robust irrespective of the program, we also simulated our bead-spring model (nucleosomes as angle-inducing proteins) using the LAMMPS software [6]. The simulations were performed with periodic boundary conditions. First we converted our parameter values into LAMMPS "real" units. The mass of DNA bead was taken 6000 g/mole and mass of octamer was taken as 55000 g/mole. In our coarse-grained model, the mass of effective nucleosome bead is equal to sum of 14 DNA beads and octamer. Simulations were performed at T=$300K$ using NVT dynamics, where Brownian-dynamics time step (Δt) was taken as $2000fs$. Other parameters are as mentioned earlier (see S1 Table).

6. BD simulation with H1 as a separate bead

To test whether the precise model for linker histone would affect our results, we did a set of simulations considering linker histone as a separate bead that interacts with two entry/exit linker DNA and the corresponding nucleosomes as suggested by data in the literature [7]. The linker springs are replaced by an H1 beads of radius a, and each H1 bead has three harmonic interactions given by $U_l = \frac{k_l}{2} \sum_i (|\mathbf{r}_i^{(1)} - \mathbf{r}^{(l)}| - 2a)^2$ where i stands for 3 DNA bead positions at the entry site, exit sites and the central DNA bead (wrapped around histone) along the dyad axis. The k_l value is taken as $30k_{\mathrm{B}}T/4a^2$. The results form this simulation is shown in S5 Fig. We have tried a few different parameters for which the results are similar.

References

[1] Allain FHT, Yen YM, Masse JE, Schultze P, Dieckmann T, Johnson RC, et al. Solution structure of the HMG protein NHP6A and its interaction with DNA reveals the structural determinants for non-sequence-specific binding. The EMBO Journal. 1999;18(9):2563–2579.

[2] Yen YM, Li X, Case DA, Giese K, Crosschedl R, Giese K, et al. Determinants of DNA binding and bending by the Saccharomyces cerevisiae high mobility group protein NHP6A that are important for its biological activities. Role of the unique N terminus and putative intercalating methionine. J Biol Chem. 1998;273(8):4424–4435.

[3] Kugel JF. Using FRET to measure the angle at which a protein Bends DNA: TBP binding a TATA box as a model system. Biochem Mol Biol Educ. 2008;36(5):341–346.

[4] Love JJ, Wong B, Johnson RC, Giese K, Crosschedl R, Giese K, et al. Structural basis for DNA bending by the architectural transcription factor {LEF-1}. Nature. 1995;376(6543):791–795.

[5] Lorenz M, Hillisch A, Payet D, Buttinelli M, Travers A, Diekmann S. DNA Bending Induced by High Mobility Group Proteins Studied by Fluorescence Resonance Energy Transfer. Biochemistry. 1999;38(37):12150–12158.

[6] Plimpton S. Fast Parallel Algorithms for Short-Range Molecular Dynamics. Journal of Computational Physics. 1995;117(1):1 – 19.

[7] Zhou BR, Jiang J, Feng H, Ghirlando R, Xiao T, Bai Y. Structural Mechanisms of Nucleosome Recognition by Linker Histones. Molecular Cell. 2015;59(4):628 – 638.
