# Supplementary material for: Binding of DNA-bending non-histone proteins destabilizes regular 30-nm chromatin structure
Source: PLoS Comput Biol. 2017 Jan 30;13(1):e1005365. doi: 10.1371/journal.pcbi.1005365 (PMC5305278; doi:10.1371/journal.pcbi.1005365)
Supplement: S2 Text — This text describes definition and formula of contact probability. (PDF) [file pcbi.1005365.s002.pdf]

S2 Text. Contact probability $I(k)$

We define I(k) which is the probability that any nucleosome is in "contact" with its kth neighbor. More precisely I(k) is the probability of finding k^{th} neighbor nucleosome below a certain cut-off distance (here the cut off distance is taken as $9a = 16nm$). To compute this probability, (similar to the procedure followed in ref [1]), we first define a square matrix $D_{i,j}$ which has elements 1 or 0 and is defined by:

$$D_{i,j}(n) = \begin{cases} 1 & \text{if } |\mathbf{r}_i^{(2)} - \mathbf{r}_j^{(2)}| < 9a \\ 0 & \text{else} \end{cases} \tag{1}$$

where n is the nth configuration (obtained from simulations), and $\mathbf{r}_i^{(2)}$ and $\mathbf{r}_j^{(2)}$ are the positions of ith and j^{th} nucleosome. Let $\overline{D}(i,j)$ be the average of this matrix over different configurations (n), in stead-state. Then we compute I(k) which is nothing but the probability of kth neighbor nucleosome below a cut-off distance of $9a$, as:

$$I(k) = \frac{\tilde{I}(k)}{\sum_j \tilde{I}(j)} \tag{2}$$

where $\tilde{I}(k) = \sum_{i=1}^{M-k} \overline{D}(i, i+k)$ and M is total number of nucleosomes present in the chromatin.

References

[1] Perisic O, Collepardo-Guevara R, Schlick T. Modeling studies of chromatin fiber structure as a function of DNA linker length. J Mol Biol. 2010;403(5):777–802.
