# Supplementary material for: Binding of DNA-bending non-histone proteins destabilizes regular 30-nm chromatin structure
Source: PLoS Comput Biol. 2017 Jan 30;13(1):e1005365. doi: 10.1371/journal.pcbi.1005365 (PMC5305278; doi:10.1371/journal.pcbi.1005365)
Supplement: S3 Text — (PDF) [file pcbi.1005365.s003.pdf]

S3 Text. Probability distribution formula for variable DNA linker length

Since linker length is not a constant across the genome [1], we did a study (FRC simulation) taking a non-uniform (variable) linker length between 31.5 bp to 73.5 bp. We assumed that length of linker DNA (L_l) is distributed according to the formula

$$P(l) = L_0 \mathrm{e}^{-(L_l/<L_l>)} \tag{1}$$

where $< L_l >$ is the average linker length and L_0 is the length constant calculated by relation $\sum_{L_l=31.5}^{73.5} P(L_l) = 1$. The results are shown S12 Fig.

References

[1] Prunell A, Kornberg RD. Variable center to center distance of nucleosomes in chromatin. J Mol Biol. 1982;154(3):515–523.
