# Supplementary material for: Binding of DNA-bending non-histone proteins destabilizes regular 30-nm chromatin structure
Source: PLoS Comput Biol. 2017 Jan 30;13(1):e1005365. doi: 10.1371/journal.pcbi.1005365 (PMC5305278; doi:10.1371/journal.pcbi.1005365)
Supplement: S4 Text — (PDF) [file pcbi.1005365.s004.pdf]

S4 Text. Chromatin: an irregular structure with fractal nature?

In vivo (and *in situ*) experiments and various analysis of such experiments suggest that chromatin has an irregular structure [1, 2, 3, 4]. Some groups have also pointed out that, on the longer length-scale, the chromatin organization has a fractal nature [5, 4, 6]. In this context, a relevant question is, does the zig-zag chromatin have a fractal nature? Another important question is what is the nature of chromatin when non-histone proteins are bound? To answer these questions we computed the root mean square of end-to-end distance ($R_{\rm rms}$) of higher-order chromatin as a function of the total length (contour length) of the 10 nm chromatin fiber. We computed end-to-end distance $R_{\rm rms}$ for different values of N in the range $10 - 10000$, where N is the total number of links (vectors or steps) in an FRC configuration. The results are shown in S14 Fig. For the case with no protein (filled squares), for small N, $R_{\rm rms} = \sqrt{\langle R^2 \rangle} \propto N$ (slope in the log-log plot is 1). Beyond hundreds of FRC steps, the curve changes its behavior and becomes $R_{\rm rms} = \sqrt{\langle R^2 \rangle} \propto N^{0.5}$—slope in the log-log plot is 0.5 indicating an ideal chain behavior. If we convert the corresponding FRC length to realistic chromatin length, one finds the linear behavior persists upto a DNA contour length of a few microns. When we introduce non-histone proteins bending the linker DNA, the scaling behavior changes (filled circles). With $\approx 30\%$ of the linker regions bound by proteins, we see that $R_{\rm rms} = \sqrt{\langle R^2 \rangle} \propto N^{0.5}$ even for relatively small N (corresponding contour length $\approx$ a few hundred nm). This implies that in the presence of non-histone protein-binding, the chromatin is highly irregular, and has a fractal nature even at short length scales.

If we compute the packing ratio, at high non-histone protein densities, we have achieved 2000 nucleosomes in a volume of $0.1(\mu m)^3$, where the volume is $\approx (4/3)\pi R_g^3$ (that is, $\approx 2000 \times 200 bp = 4 \times 10^5$ bp in 0.1 cubic micron). Even though this is not as high as organisms like Paris Japonica, this is comparable (or slightly higher than) a simple human chromatin estimate (3×10^9 bp in $(10\mu m)^3$).

If the chain is made non-ideal, the slope is expected to change appropriately; however, the random/irregular nature will remain. One may also extend this study to investigate statistics of loops and loop length distributions in the context of longer chromatin [5]; however, since other interactions (e.g., lamin proteins) may influence the result, we are deferring it at present.

References

[1] Bouchet-Marquis C, Dubochet J, Fakan S. Cryoelectron microscopy of vitrified sections: a new challenge for the analysis of functional nuclear architecture. Histochem Cell Biol. 2006;125(1-2):43–51.

[2] Eltsov M, Maclellan KM, Maeshima K, Frangakis AS, Dubochet J. Analysis of cryo-electron microscopy images does not support the existence of 30-nm chromatin fibers in mitotic chromosomes in situ. Proc Natl Acad Sci U S A. 2008;105(50):19732–19737.

[3] Grigoryev SA, Arya G, Correll S, Woodcock CL, Schlick T. Evidence for heteromorphic chromatin fibers from analysis of nucleosome interactions. Proc Natl Acad Sci U S A. 2009;106(32):13317–13322.

[4] Nishino Y, Eltsov M, Joti Y, Ito K, Takata H, Takahashi Y, et al. Human mitotic chromosomes consist predominantly of irregularly folded nucleosome fibres without a 30-nm chromatin structure. EMBO J. 2012;31(7):1644–1653.

[5] Lieberman-Aiden E, van Berkum NL, Williams L, Imakaev M, Ragoczy T, Telling A, et al. Comprehensive Mapping of Long-Range Interactions Reveals Folding Principles of the Human Genome. Science. 2009;326(5950):289–293.

[6] Bancaud A, Huet S, Daigle N, Mozziconacci J, Beaudouin J, Ellenberg J. Molecular crowding affects diffusion and binding of nuclear proteins in heterochromatin and reveals the fractal organization of chromatin. EMBO journal. 2009;28(24):3785–3798.
