# Supplementary material for: Binding of DNA-bending non-histone proteins destabilizes regular 30-nm chromatin structure
Source: PLoS Comput Biol. 2017 Jan 30;13(1):e1005365. doi: 10.1371/journal.pcbi.1005365 (PMC5305278; doi:10.1371/journal.pcbi.1005365)
Supplement: S1 Table — (PDF) [file pcbi.1005365.s005.pdf]

S1 Table. BD parameter description

Parameter	Description	Value	Reference or rationale
T	Temperature	300 K	[1]
$2a$	Diameter of type 1 bead	3.4 nm	One helix turn of DNA which is 10.5 bp (3.4 nm) [1]
r_s	Equilibrium distance for DNA chain	3.4 nm	[1]
r_n	Equilibrium distance for nucleosome core-DNA interaction	$8r_s/\pi$	DNA is wrapped around histone surfaces with 14 contact point makes 1.75 turns. so $r_n = 14r_s/(1.75 * \pi)$
r_l	Equilibrium distance for linker histone interaction	$2.5r_s$	Depends on the nucleosome angle. We varied this parameter
r_p	Equilibrium distance for non-histone protein binding at the linker region	$1.5r_s$	Depends on the non-histone protein bending angle. We varied this.
r_h	Equilibrium distance for inter-nucleosome interaction	$4.2r_s$	There are 4 type of histones H2A, H2B, H3 and H4. The average length their tails is: 7.8 nm [1]. So cut-off is $2 * 7.8 * r_s/3.4 = 4.58r_s$. So we took 90% of the cut-off.
k_s	Stretching stiffness for DNA chain	$100k_BT/r_s^2$	For the purpose of this work, the exact value of this spring stiffness parameter is irrelevant as long as it is high enough to keep the spring unstretchable. We have used a high value such that the interaction is stable at its equilibrium length.
k_n	Stretching stiffness for nucleosome core-DNA interaction	$100k_BT/r_s^2$	For the purpose of this work, the exact value of this spring stiffness parameter is irrelevant as long as it is high enough to keep the spring unstretchable. We have used a high value such that the interaction is stable at its equilibrium length.
k_l	Stretching stiffness for linker histone interaction	$30 - 100k_BT/r_s^2$	For the purpose of this work, the exact value of this spring stiffness parameter is irrelevant as long as it is high enough to keep the spring unstretchable. We have used a high value such that the interaction is stable at its equilibrium length.
k_p	Stretching stiffness for non-histone protein binding at the linker region	$100k_BT/r_s^2$	For the purpose of this work, the exact value of this spring stiffness parameter is irrelevant as long as it is high enough to keep the spring unstretchable. We have used a high value such that the interaction is stable at its equilibrium length.
k_b	Bending stiffness of type 1 bead	$50k_BT$	[2, 3]
ϵ	LJ parameter	k_BT	[4]
$\mu_0^{(1)}$	Mobility of type 1 bead	$.0002r_s/k_BT\Delta t$	[4]
$\mu_0^{(2)}$	Mobility of type 2 bead	$.00015r_s/k_BT\Delta t$	$\tilde{\mu}_0 \propto 1/(\text{radius of bead})^3$
Δt	Time step in which event occurs	0.04 ns	Using relation $\tilde{\mu}_0 = \mu_0 k_BT\Delta t/4a^2$ [4]

References

[1] Perisic O, Collepardo-Guevara R, Schlick T. Modeling studies of chromatin fiber structure as a function of DNA linker length. J Mol Biol. 2010;403(5):777–802.

[2] Yan J, Kawamura R, Marko JF. Statistics of loop formation along double helix DNAs. Phys Rev E Stat Nonlin Soft Matter Phys. 2005;71(6 Pt 1):061905.

[3] Ranjith P, Kumar PBS, Menon GI. Distribution Functions, Loop Formation Probabilities, and Force-Extension Relations in a Model for Short Double-Stranded DNA Molecules. Phys Rev Lett. 2005;94(13):138102.

[4] Netz R. Nonequilibrium Unfolding of Polyelectrolyte Condensates in Electric Fields. Phys Rev Lett. 2003;90(12):128104.
