# Supplementary material for: Binding of DNA-bending non-histone proteins destabilizes regular 30-nm chromatin structure
Source: PLoS Comput Biol. 2017 Jan 30;13(1):e1005365. doi: 10.1371/journal.pcbi.1005365 (PMC5305278; doi:10.1371/journal.pcbi.1005365)
Supplement: S2 Table — (PDF) [file pcbi.1005365.s006.pdf]

S2 Table. Non-dimensional parameters

$\tilde{U}$	Energy	U/k_BT
$\tilde{\mathbf{r}}$	positions	$\mathbf{r}/2a$
$\tilde{k}_\alpha$	stretching energy constants	$2k_\alpha a^2/k_BT$
$\tilde{k}_b$	bending stiffness	k_b/k_BT2a
$\tilde{\mu}_0$	mobility	$\mu_0 k_BT\Delta t/4a^2$
n	time step	$t/\Delta t$
